# Silibinin Regulates Tumor Progression and Tumorsphere Formation by Suppressing PD-L1 Expression in Non-Small Cell Lung Cancer (NSCLC) Cells

**DOI:** 10.3390/cells10071632

**Published:** 2021-06-29

**Authors:** Alexis Rugamba, Dong Young Kang, Nipin Sp, Eun Seong Jo, Jin-Moo Lee, Se Won Bae, Kyoung-Jin Jang

**Affiliations:** 1 Department of Pathology, Institute of Biomedical Science and Technology, School of Medicine, Konkuk University, Chungju 27478, Korea; ra0909@konkuk.ac.kr (A.R.); kdy6459@kku.ac.kr (D.Y.K.); nipinsp@konkuk.ac.kr (N.S.); 2 Pharmacological Research Division, National Institute of Food and Drug Safety Evaluation, Osong Health Technology Administration Complex, Cheongju-si 28159, Korea; eses0706@korea.kr (E.S.J.); elzem@korea.kr (J.-M.L.); 3 Department of Chemistry and Cosmetics, Jeju National University, Jeju 63243, Korea; swbae@jejunu.ac.kr

**Keywords:** silibinin, EGFR, JAK2/STAT5b, PI3K/AKT, MMP2, PD-L1, NSCLC, tumorsphere

## Abstract

Recently, natural compounds have been used globally for cancer treatment studies. Silibinin is a natural compound extracted from *Silybum marianum* (milk thistle), which has been suggested as an anticancer drug through various studies. Studies on its activity in various cancers are undergoing. This study demonstrated the molecular signaling behind the anticancer activity of silibinin in non-small cell lung cancer (NSCLC). Quantitative real-time polymerase chain reaction and Western blotting analysis were performed for molecular signaling analysis. Wound healing assay, invasion assay, and in vitro angiogenesis were performed for the anticancer activity of silibinin. The results indicated that silibinin inhibited A549, H292, and H460 cell proliferation in a concentration-dependent manner, as confirmed by the induction of G0/G1 cell cycle arrest and apoptosis and the inhibition of tumor angiogenesis, migration, and invasion. This study also assessed the role of silibinin in suppressing tumorsphere formation using the tumorsphere formation assay. By binding to the epidermal growth factor receptor (EGFR), silibinin downregulated phosphorylated EGFR expression, which then inhibited its downstream targets, the JAK2/STAT5 and PI3K/AKT pathways, and thereby reduced matrix metalloproteinase, PD-L1, and vascular endothelial growth factor expression. Binding analysis demonstrated that STAT5 binds to the PD-L1 promoter region in the nucleus and silibinin inhibited the STAT5/PD-L1 complex. Altogether, silibinin could be considered as a candidate for tumor immunotherapy and cancer stem cell-targeted therapy.

## 1. Introduction

Tumor progression is the final phase in tumor development characterized by the increased growth rate and invasiveness of tumor cells. Furthermore, this change causes the tumor to become more aggressive and acquires greater malignant potential [1]. This process may cause the increase of particular proteins that contribute to tumor metastasis and invasion [2] and lead to the formation of new micro vessels, a process called angiogenesis [3]. Moreover, another pathogenic character of cancer is found in non-small cell lung cancer (NSCLC), that is, hepatocyte growth factor promotes cancer stem cell (CSC) sphere formation [4]. Tumorsphere formation has recently been used as a key property of cancer cells to evaluate these CSC properties, and a sphere formation assay has been introduced as a specific approach to study adult stem cells [5]. In non-adherent plates with free serum medium in nutrition-deficient conditions, CSCs will proliferate and form a sphere, whereas differentiated tumor cells undergo cell death [5,6]. Suppression of tumorsphere formation could lead to the inhibition of tumor growth.

Different signaling pathways, such as phosphatidylinositol 3-kinase (PI3K)/AKT and JAK/STAT, have been identified to play an important role in cancer [7]. Despite its great functions in the cell cycle, such as cell growth, metabolism, and survival, PI3K/AKT is another pathway that may also involve tumor progression by connecting to vascular endothelial growth factor (VEGF), resulting in angiogenesis [8,9]. This is considered an important signaling pathway due to its role in tumor invasion by regulating matrix metalloproteinases (MMPs) through mammalian target of rapamycin signaling [10]. PI3K/AKT signaling is a key target for anti-metastatic therapy against tumor metastasis of esophageal squamous cell carcinoma [11], and it could also regulate tumor invasion and metastasis by mediating MMP expressions [12].

The JAK-STAT pathway follows usual signaling models where the ligand attaches to the extracellular side of a transmembrane receptor and activates a cascade of phosphorylation responses. These reactions consist of phosphorylation of JAK kinases, the receptor cytoplasmic tail, and STAT transcription factors that phosphorylate STATs and cause localization to the nucleus, DNA binding, and gene regulation [13]. Thus, there are many approaches focused on developing therapy through the suppression of STAT5 phosphorylation, and the beneficial effects of its activity reduction in cancer have been clearly demonstrated in vitro and in vivo [14]. MMPs have a connection with the JAK/STAT pathway in playing a key role in cancer progression through invasion, angiogenesis, and migration [15]. Its control has been demonstrated as one of the potential tools that can be used to prevent tumor invasion and thereby cancer progression [16].

PD-1/PD-L1 is a complex that plays a pivotal role in tumor progression by its involvement in growth regulation disturbance, resulting in a defect in programmed cell death or apoptosis [17]. In addition, a high level of PD-L1 has been confirmed to appear on the surface of different cancer cells, including NSCLC, justifying that its expression facilitates cancer cells to escape immune response [18]. PI3K/AKT and JAK/STAT pathways play a vital role in the expression of PD-L1 in tumor cells which takes part in different cancer hallmarks [19,20]. The PD-1/PD-L1 inhibitory checkpoint suppresses antitumor immune responses [21,22]. Many studies have been carried out, and findings on anti-PD-1/PD-L1 agents that inhibit its signaling have indicated good and promising activity in metastatic NSCLC [23,24].

Many natural compounds are known to have anticancer activity and have been found to be effective against pathogenesis for a long time [25]. Silibinin is a natural compound extracted from *Silybum marianum* (milk thistle), a plant traditionally used to manage liver diseases, and studies on silibinin have indicated a significant antineoplastic activity in various cancer types, such as lung, skin, breast, kidney, colon, and prostate [26]. The potential use of silibinin in novel anticancer therapy has received considerable interest in the past few years. Whereas many studies have described the mechanistic events associated with various cell signaling pathways [26], silibinin has also shown an effective role in the management of lung cancer through the inhibition of proliferation and angiogenesis [27]. Hence, researchers have suggested further studies on the effects of silibinin on lung cancer.

Therefore, this study focused more on the inhibition of cancer hallmarks by silibinin in lung cancer by checking the expression levels of target proteins in three types of NSCLC cells (A549, H292, and H460).

## 2. Materials and Methods

### 2.1. Antibodies and Cell Culture Reagents

RPMI-1640 medium, 0.05% trypsin-EDTA, and penicillin-streptomycin solution were obtained from Gibco (Thermo Fisher Scientific, Inc., Waltham, MA, USA). Silibinin and fetal bovine serum (FBS) were purchased from Sigma-Aldrich (Merck KGaA, St. Louis, MO, USA). Antibodies specific for VEGF (sc-507), STAT5b (sc-1656), CDK4 (sc-260), MMP9 (sc-13520), cyclin E (sc-481), and glyceraldehyde 3-phosphate dehydrogenase (GAPDH; #2118) together with their specific secondary antibodies (anti-mouse (sc-516102) and anti-rabbit (sc-2357)) were purchased from Santa Cruz Biotechnology (Dallas, TX, USA). Antibodies specific for p27 (#3686), p21 (#2974), phosphorylated epidermal growth factor (EGF) receptor (pEGFR; #3777), EGFR (#4267), phosphorylated JAK2 (pJAK2; (#3776) and JAK2 (#3230)), and phosphorylated STAT5 (pSTAT5; #9351) were purchased from Cell Signaling Technology (Beverly, MA, USA). Cyclin D1 (ab6152) antibody was obtained from Abcam (Cambridge, MA, USA). SOX2 (#MAB4423), NANOG (#MABD24), OCT4 (#MABD76), and MMP3 (#AB2963) were purchased from Merck Millipore (Burlington, MA, USA). The MMP2 (E90317) antibody was obtained from EnoGene (New York, NY, USA). The antibody specific for PD-L1 (R30949) was purchased from NSJ Bioreagents (San Diego, CA, USA).

### 2.2. Cell Culture and Treatment

A549 (no. 10185; Korean Cell Line Bank, Seoul, Korea), H460 (no. 30177; Korean Cell Line Bank), and H292 (no. 21848; Korean Cell Line Bank) cell lines were maintained in RPMI-1640 plus 10% FBS and 1% penicillin at 37 ℃ in 5% CO_2_. The medium was changed thrice a week after the cells were grown up to 80% confluence and then treated with silibinin and incubated at 37 ℃ for 48 h.

### 2.3. Cell Viability Assay

Cell viability was measured using the 3-(4,5-dimethylthiazol-2-yl)-2,5-diphenyltetrazolium bromide (MTT) assay. Cells were maintained in RPMI-1640 in 96-well culture plates at 3 × 10^3^ per well (density) for 24 h. Cells were again incubated with a new medium containing dimethyl sulfoxide (DMSO) as vehicle control and then treated with various silibinin concentrations (50–400 μM) for 48 h. MTT (5 mg/mL) was added and again incubated for 4 h at 37 ℃. The resulting formazan product was dissolved in DMSO, and an Ultra Multifunctional Microplate Reader (Tecan, Durham, NC, USA) was used to measure the absorbance at a wavelength of 560 nm. All measurements were performed in triplicate, and experiments were repeated thrice. 

### 2.4. 4′,6-diamidino-2-phenylindole (DAPI) Staining and Morphological Analysis

The DAPI staining technique was used to examine apoptotic condensed chromatin. A549, H292, and H460 cells were cultured using six-well plates at a density of 1.5 × 10^5^ per well and treated with 50 and 100 μM silibinin for 48 h, followed by two-time washing with phosphate-buffered saline (PBS), and again incubated with 500 μL of 300 nM DAPI staining solution for 1 h. Cells were then washed twice with PBS. Stained cells were mounted using a mounting solution on microscope slides. Images were captured using a fluorescence microscope (Olympus IX71/DP72, Tokyo, Japan).

### 2.5. Western Blotting Analysis

Protein samples were isolated from untreated (control) or silibinin-treated H292, A549, and H460 cells using radioimmunoprecipitation lysis buffer (20-188; EMD Millipore) containing phosphatase and protease inhibitors. The concentration was measured using Bradford’s method (Thermo Fisher Scientific). Equal amounts of protein (100 μg/well) were dissolved with 10% to 15% sodium dodecyl sulfate-polyacrylamide gel electrophoresis. Separated proteins were then transferred onto nitrocellulose membranes. The blots were blocked for 1 h with 5% skim milk (BD Biosciences, San Jose, CA, USA) in TBS-T buffer (20 mM Tris-HCl (Sigma-Aldrich; Merck KGaA), pH 7.6, 137 mM NaCl (Formedium, Norfolk, UK; NAC03), and 0.1× Tween 20 (Scientific Sales, Inc., Oak Ridge, TN, USA)). The membranes were then incubated overnight at 4 °C in a shaker with primary antibodies diluted in 5% bovine serum albumin (EMD Millipore). The membranes were then washed with TBS-T and incubated for 1 h at room temperature with horseradish peroxidase-conjugated secondary antibodies. Detection was performed using a Femto Clean Enhanced Chemiluminescence Solution Kit (77449; GenDEPOT, Katy, TX, USA) and a LAS-4000 imaging device (Fujifilm, Tokyo, Japan).

### 2.6. Quantitative Real-Time Polymerase Chain Reaction (qPCR)

The RNeasy Mini Kit (Qiagen GmbH, Hilden, Germany) was used to extract total RNA. Total RNA was then quantified using a spectrophotometer at 260 nm. A Thermal cycler (C1000 Thermal Cycler; Bio-Rad, Hercules, CA, USA) was used to make cDNA from the total RNA at 42 °C for 1 h and 95 °C for 5 min using a first-strand cDNA synthesis kit (Bioneer, Daejeon, Korea) and oligo (dT) primers. MMP2, MMP3, MMP9, VEGF, NANOG, SOX2, OCT4, and GAPDH cDNA were amplified using the RT-PCR Premix Kit (Bioneer) with primers synthesized by Bioneer. A LightCycler 480II (Roche) was used for qPCR as follows: 2 μL diluted cDNA was mixed with 1 μL forward and 1 μL reverse primers and 10 μL TB Green Advantage Premix (Takara Bio, Japan). The cycling conditions were as follows: 5 min for initial denaturation at 95 °C, followed by 40 cycles of 40 s at 95 °C, 40 s at 58 °C, 40 s at 72 °C, and 5 min at 72 °C. All reactions were performed in triplicate and normalized to GAPDH. The primers are listed in Supplementary Table S1. The calculations were carried out using the Cp values.

### 2.7. Cell Cycle Analysis

After A549, H292, and H460 cells were treated with silibinin, the DNA content of non-treated and treated cells was determined by a BD Cycle Test Plus DNA Reagent Kit (BD Biosciences) according to the manufacturer’s protocols. About 5 × 10^5^ cells were treated with or without silibinin for 48 h, washed with PBS, and permeabilized with trypsin. After staining with propidium iodide (PI), cells were analyzed by flow cytometry through FACSCalibur (BD Biosciences).

### 2.8. Apoptosis Analysis

Fluorescein isothiocyanate (FITC)-conjugated Annexin V (Annexin V-FITC) was used to measure apoptosis in A549, H292, and H460 cells. After washing with PBS, silibinin-treated cells were re-suspended in a binding buffer (1 × 10^6^ cells/mL) and stained with Annexin V-FITC and PI for 10 min in the dark at room temperature. The apoptotic cell percentage was measured using flow cytometry through FACSCalibur and analyzed using FlowJo software.

### 2.9. In Vitro Angiogenesis Assay

The in vitro angiogenesis kit was ordered from Millipore (Billerica, MA, USA). The EC Matrix was thawed at 4 °C overnight. The required wells of the pre-chilled 96-well plates were coated with diluted EC Matrix (50 μL) and incubated at 37 °C for 1 h to solidify. Human umbilical vein endothelial cells (HUVECs; 1 × 10^4^; 150 μL) with or without silibinin were added to the solidified matrix and incubated at 37 °C for 8 h. Endothelial cell formation was observed using a microscope. The focus was placed on distinct areas, and the tubes formed were counted according to the kit procedure.

### 2.10. Matrigel Invasion Assay

Precoated Matrigel was used to conduct a Transwell invasion assay using invasion chambers (BD Biocoat, Bedford, MA, USA). Cells (5 × 10^4^) were added to the inserts, whereas both medium and drug were added to the receiver plate. The inserts containing cells were retained onto the receiver plate and incubated at 37 °C in a humidified chamber for 48 h. After incubation, cells that invaded the apical surface of the inserts were fixed with crystal violet, whereas those that stayed attached to the upper surface of the insert were removed using a cotton swab. The invaded cells were observed using a microscope and counted.

### 2.11. Wound Healing Assay

A549, H292, and H460 cells were seeded in six-well plates at 1 × 10^5^ per well and incubated in RPMI-1640 for 24 h. After becoming a confluent monolayer, the cell layers were scratched with a pipette tip and washed with PBS to remove cell debris and immediately treated with silibinin for 48 h, except control cells. Using microscopy, photos were taken at different time intervals to evaluate the wound edges, and the relative area of wound closure was measured using ImageJ software (NIH Image, Bethesda, MD, USA).

### 2.12. Tumorsphere Formation Assay

A549, H292, and H460 cells were incubated in DMEM/F-12 containing growth supplements, EGF, basic fibroblast growth factor (bFGF), and B27 in low attachment six-well plates together with silibinin for 14 days. Photos were taken on days 0, 7, and 14 using a microscope. Total RNA was extracted from the sphere and analyzed using qPCR.

### 2.13. STAT5 and AKT1/2 Inhibitors

A549, H292, and H460 cells (1 × 10^6^) were seeded in 6 cm plates and grown to 60% confluence and then treated with the STAT5 inhibitor (573108-10MG; Sigma-Aldrich; Merck KGaA) for 6 h. Cells were also treated with the AKT1/2 inhibitor (A6730-5MG; Sigma-Aldrich; Merck KGaA) for 1 h, followed by the treatment with or without silibinin for another 48 h under cell culture conditions. Proteins were isolated and analyzed using Western blotting.

### 2.14. Chromatin Immunoprecipitation (ChIP) Assay

ChIP assay was performed using an Imprint ChIP Kit (Sigma-Aldrich) according to the manufacturer’s protocol. A549, H292, and H460 cells were fixed with 1% formaldehyde, quenched with 1.25 M glycine, washed with PBS, suspended in a nuclear preparation and shearing buffer, and then sonicated. Sheared DNA was centrifuged, followed by protein/DNA immunoprecipitation from the cleared supernatant as follows. The clarified supernatant was diluted with buffer (1:1 ratio), and 5 µL aliquots of the diluted samples were used as internal controls. Next, the diluted supernatant was incubated with anti-STAT5b antibodies in precoated wells for 90 min. The controls were incubated with normal goat IgG and anti-RNA polymerase II. Unbound DNA was removed, and bound DNA was collected using the cross-link reversal method with DNA release buffer containing proteinase K. The released DNA and internal control DNA were purified using the GenElute Binding Column G. DNA was then quantified using specific PD-L1 primers (Supplementary Table S1) by qPCR.

### 2.15. Statistical Analyses

All experiments were performed at least thrice. The results are expressed as the mean ± standard error of the mean (SEM). Statistical analyses were conducted via one-way analysis of variance (ANOVA) or Student’s *t*-test. One-way ANOVA was performed with Tukey’s post hoc test. The analyses were performed using SAS version 9.3 software program (SAS Institute, Inc., Cary, NC). *p* < 0.05 (*) indicates a significant difference.

## 3. Results

### 3.1. Silibinin Prevents NSCLC Cell Proliferation and Induces Cell Cycle Arrest and Apoptosis

**Figure 1:**
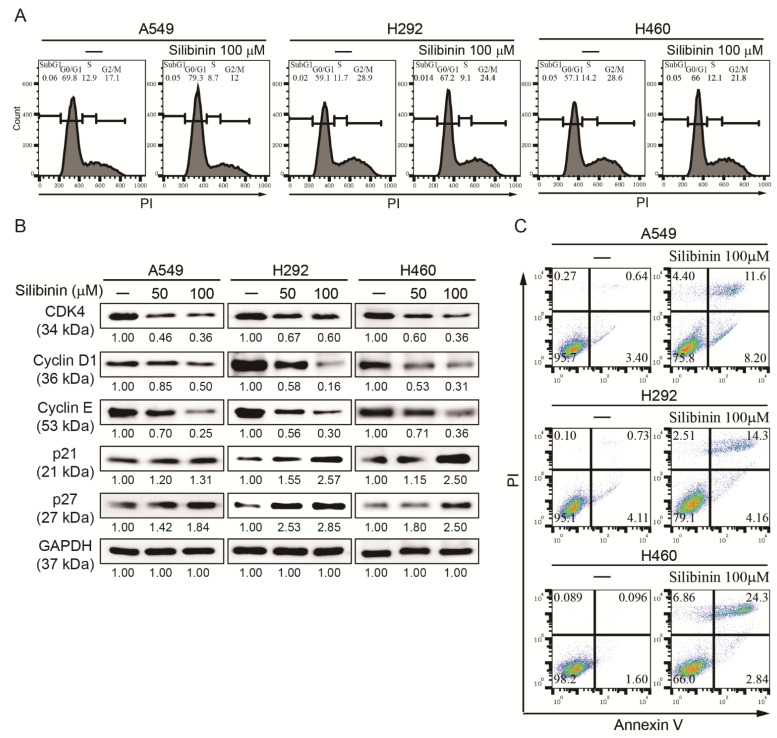
Silibinin caused apoptosis and cell cycle arrest in NSCLC. (**A**) Cell cycle analysis with flow cytometry using PI staining showed cell cycle distribution and G0/G1 arrest by 100 µM silibinin. (**B**) Western blotting analysis of A549, H292, and H460 cells with 50 and 100 µM silibinin for 48 h showed suppression of CDK4, cyclin D1, and cyclin E and elevation of p21 and p27 protein expression. GAPDH was used as a housekeeping gene. Controls were set to 100. Experiments were repeated thrice to confirm the data. (**C**) Flow cytometry analysis using Annexin V/PI staining after treatment with 100 µM silibinin for 48 h, indicating early and late apoptosis in A549 and H292 cells and only late apoptosis in H460 cells.

It was hypothesized that silibinin could induce a concentration-dependent cell death against NSCLC. MTT assay analysis indicated that exposure of A549, H292, and H460 cells to different concentrations of silibinin (50, 100, 200, 300, and 400 µM) for 48 h caused a significant decrease in cell viability, where 50% inhibition was marked at a concentration of 100 µM (Figure S1A). Microscopic observation with the DAPI staining solution showed that the cell number was reduced depending on the concentration of silibinin, indicating the anticancer activity of silibinin (Figure S1B). Hence, 50 and 100 µM silibinin were used for further studies. Treatment of A549, H292, and H460 with 50 and 100 µM silibinin showed an inhibitory effect of silibinin on NSCLC, suggesting that silibinin may have the ability to induce cell cycle arrest and apoptosis. Using flow cytometry, a cell cycle arrest in the G0/G1 phase by 100 µM silibinin was found in all three NSCLC cell lines (Figure 1A). To confirm cell cycle arrest induction by silibinin, Western blotting was performed to determine the downregulation of cell cycle marker protein (CDK4, cyclin D1, and cyclin E) expression and upregulation of the expression level of tumor suppressor proteins (p21 and p27; Figure 1B). Induction of cell cycle arrest suggested a possible induction of cell apoptosis induction by silibinin. Therefore, the apoptosis level was checked using flow cytometry, and an increase in cell death by 100 µM silibinin was observed (Figure 1C). These results suggested the anticancer activity of silibinin against NSCLC cells.

### 3.2. Silibinin Inhibits Angiogenesis of HUVEC Cells

**Figure 2:**
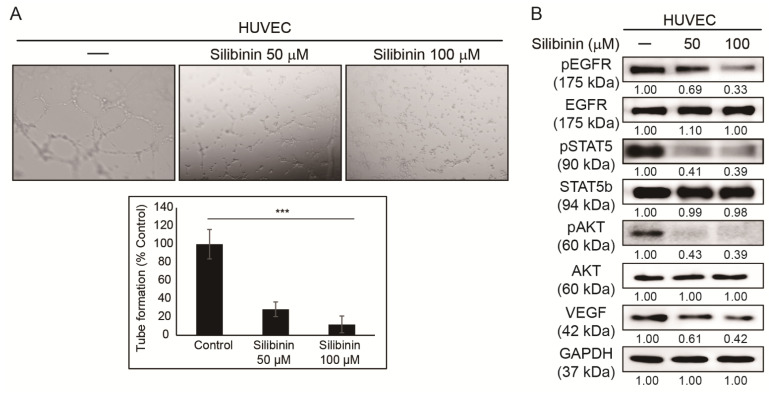
Silibinin inhibited angiogenesis. (**A**) In vitro angiogenesis assay indicated that 50 and 100 µM silibinin in 48 h reduced tube formation in HUVECs compared to the control. (**B**) Western blotting analysis after HUVECs were treated with 50 and 100 µM silibinin for 48 h showing suppressing activity in pEGFR, pSTAT5, pAKT, and VEGF expression. The relative protein expression level was measured using densitometry and normalized to GAPDH. Controls were set to 100. *** *p* < 0.001 (ANOVA). To prove the effectiveness of data, experiments were repeated thrice.

The ability of silibinin to induce cell cycle arrest suggests the inhibition of cancer hallmarks by silibinin. Hence, it was hypothesized that silibinin could also suppress tumor angiogenesis. HUVECs were used to evaluate in vitro angiogenesis and assess the ability of silibinin to suppress angiogenesis [28]. Angiogenesis assay was performed and showed tube formation in the control sample, whereas silibinin-treated samples showed a significant decrease in tube formation (Figure 2A). Moreover, Western blotting in HUVECs was done to confirm this effect at the protein level. This analysis indicated that silibinin has a suppressing activity on the angiogenic factor, VEGF, which confirmed the antiangiogenic activity of silibinin (Figure 2B). Downregulation in the phosphorylation of key molecular signaling EGFR, STAT5, and AKT in HUVECs by silibinin was also observed. These results suggested the possible role of PI3K/AKT and JAK/STAT5 signaling in the anticancer activity of silibinin.

### 3.3. Silibinin Inhibits NSCLC Cell Migration and Invasion

**Figure 3:**
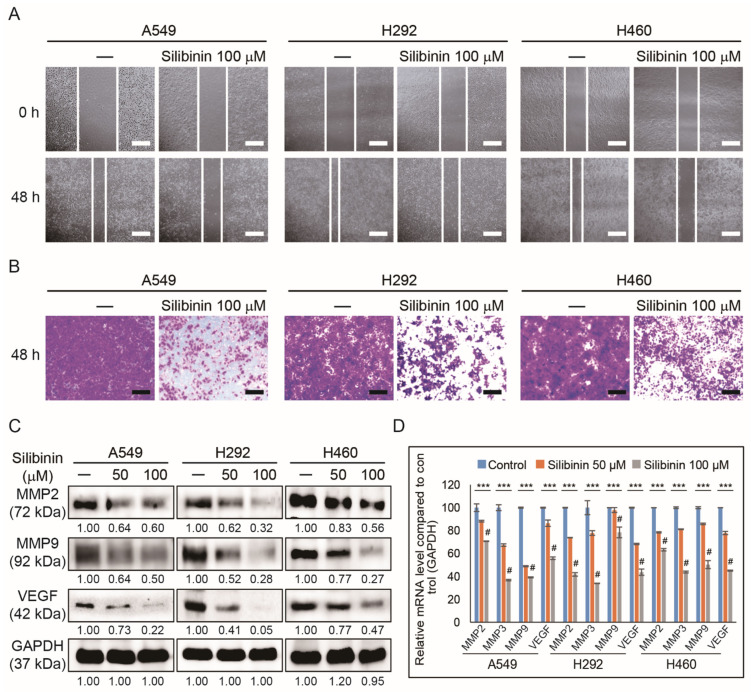
Silibinin inhibited migration and invasion. (**A**) Wound healing assay was used to assess the migration inhibition ability of 50 and 100 µM silibinin in A549, H292, and H460 cells for 0 and 48 h. Scale bar, 50 μm. (**B**) Matrigel invasion assay illustrated that 50 and 100 µM silibinin for 48 h could inhibit the invasion of A549, H292, and H460 cells. Scale bar, 100 μm. (**C**) Western blotting analysis of A549, H292, and H460 cells with 50 and 100 µM silibinin for 48 h showed a downregulation of MMP2, MMP9, and VEGF. Densitometry was used to determine the relative expression of proteins and was normalized to GAPDH. Controls were set to 100. Experiments were repeated thrice to confirm the data. (**D**) Real-time qPCR analysis showing illustrative expression of MMP2, MMP3, MMP9, and VEGF genes in A549, H292, and H460 cells. Cp values were normalized to *GAPDH* mRNA. Controls were set to 100. *** *p* < 0.001 (ANOVA). # *p* < 0.001 vs. control.

The inhibition of angiogenesis by silibinin against HUVEC cells was demonstrated. Therefore, it was assumed that silibinin might have the ability to suppress tumor metastasis. To evaluate metastasis, the ability of silibinin to inhibit tumor migration and invasion was analyzed. To identify whether NSCLC cells could migrate after exposure to silibinin, a wound healing assay was performed, indicating a migration inhibitory effect in all three cell lines (A549, H292, and H460; Figure 3A). An invasion assay was also done to check whether silibinin can suppress tumor metastasis, and the results showed significant inhibition of invaded cells in treated NSCLC cell lines (Figure 3B). To confirm invasion inhibition ability, molecular markers were analyzed for invasion, and Western blotting analysis of MMP2, MMP9, and VEGF also confirmed that silibinin could reduce angiogenesis, progression, and metastasis of NSCLC (Figure 3C).

### 3.4. Silibinin Suppresses Cancer Stemness by Tumorsphere Inhibition in NSCLC Cells

**Figure 4:**
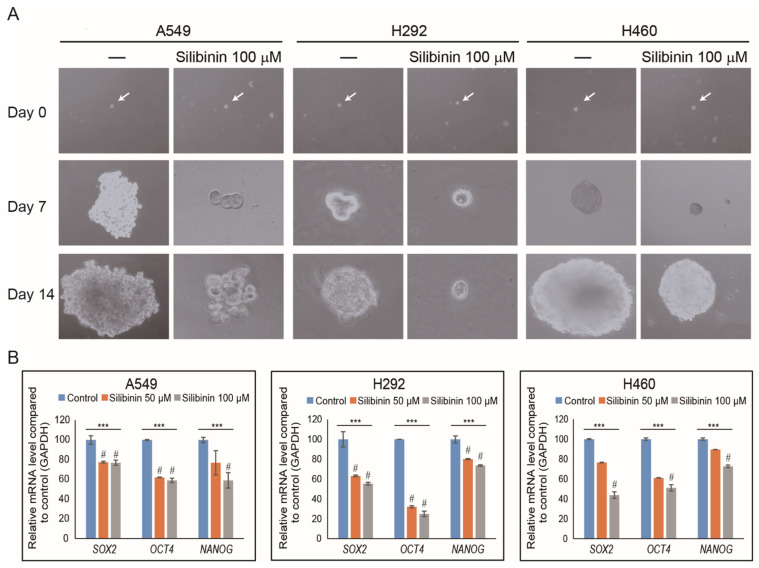
Silibinin inhibited tumorsphere formation in NSCLC cells. (**A**) A549, H292, and H460 cells were cultured in DMEM/F-12 containing EGF, bFGF, and B27 for 14 days. Images were taken on days 0, 7, and 14 and showed that the tumorsphere increased in size in non-treated cells but reduced in those treated with silibinin. (**B**) After 14 days of incubation, mRNA was isolated from treated and non-treated (control) tumorspheres and analyzed by qPCR, which showed CSC marker genes in A549, H292, and H460 cells. The illustrative expression of *SOX2*, *OCT4*, and *NANOG* mRNA expression is shown. Cp values were normalized to *GAPDH* mRNA. Controls were set to 100. *** *p* < 0.001 (ANOVA). # *p* < 0.001 vs. control.

Although it was found that silibinin has an inhibitory effect on cancer hallmarks in NSCLC cells, its capability to inhibit CSCs has been identified in a study [29]. For the confirmation, tumorsphere formation assay was performed to evaluate the ability of silibinin on cancer stemness inhibition ability using NSCLC cells. A549, H292, and H460 cells were incubated in tumorsphere media using a low attachment plate together with silibinin for 14 days. Photos were taken using a microscope, and the images showed a significant decrease in tumorsphere by 100 µM silibinin compared to non-treated controls (Figure 4A). To confirm the inhibition of cancer stemness by silibinin, qPCR was conducted to check the expression of specific CSC markers (*SOX2*, *OCT4*, and *NANOG*), and significant inhibition of these stem cell markers by silibinin has been noted (Figure 4B). These results clearly showed the ability of silibinin to target CSCs.

### 3.5. Silibinin Inhibited EGFR Pathway and PD-L1 Expression in NSCLC Cells

**Figure 5:**
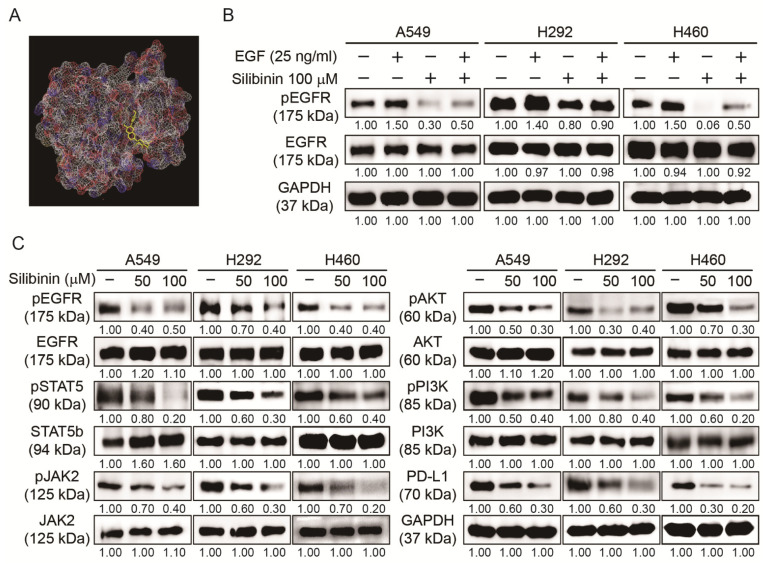
Silibinin regulated EGFR-mediated JAK2/STAT5 and PI3K/AKT signaling and PD-L1 expression. (**A**) Molecular docking using AutoDock Vina software showed silibinin binding (PubChem ID: 31553) to the ATP-binding domain of EGFR (PDB ID: 2GS2). (**B**) Western blotting analysis of A549, H292, and H460 cells pretreated with EGF (25 ng/mL) for 1 h and then treated with 100 µM silibinin for 48 h indicated the upregulation patterns of EGFR for EGF-treated samples and downregulation patterns of silibinin for silibinin/EGF-treated cells. Representative expression of proteins was determined by densitometry and normalized to GAPDH. Controls were set to 100. Experiments were repeated thrice to confirm the data. (**C**) Western blotting analysis of proteins showing the expression levels of EGFR, JAK2/STAT5, and PI3K/AKT signaling and PD-L1 expression in A549, H292, and H460 cells after exposure to 50 and 100 µM silibinin for 48 h. Representative expression of proteins was determined by densitometry and normalized to GAPDH. Controls were set to 100. Experiments were repeated thrice to confirm the data.

After confirming that silibinin has the capacity to suppress cancer hallmarks and CSC formation, the molecular signaling behind these mechanisms was also checked by starting from its binding to EGFR. First, we examined whether silibinin could bind to the EGF receptor using molecular docking analysis and the obtained results suggested an interaction between silibinin and EGFR with a strong binding affinity of −8.3 kcal/mol, suggesting that silibinin acted through EGFR signaling (Figure 5A). The detailed mechanism on the binding of silibinin with EGFR has been already studied in 2D as well as 3D images [30]. To confirm these results, we conducted Western blotting analysis in NSCLC cells with or without recombinant human EGF or silibinin. The obtained result indicated an increase in pEGFR expression in EGF-treated cells, which was significantly reduced by 100 µM silibinin (Figure 5B). This inhibition of EGFR phosphorylation blocked cellular signaling toward the downstream targets of EGFR. This was confirmed by Western blotting, which showed the inhibitory effect of silibinin against the phosphorylation expression of JAK2/STAT5 and PI3K/AKT cell molecular signaling pathways without altering their total proteins. This inhibition ability of silibinin was also observed in PD-L1 protein expression in all three NSCLC cells (Figure 5C), indicating the role of PD-L1 in the inhibitory effects of silibinin.

### 3.6. Silibinin Impaired STAT5-Dependent PD-L1 Expression and STAT5 Binding to the PD-L1 Promoter Region

**Figure 6:**
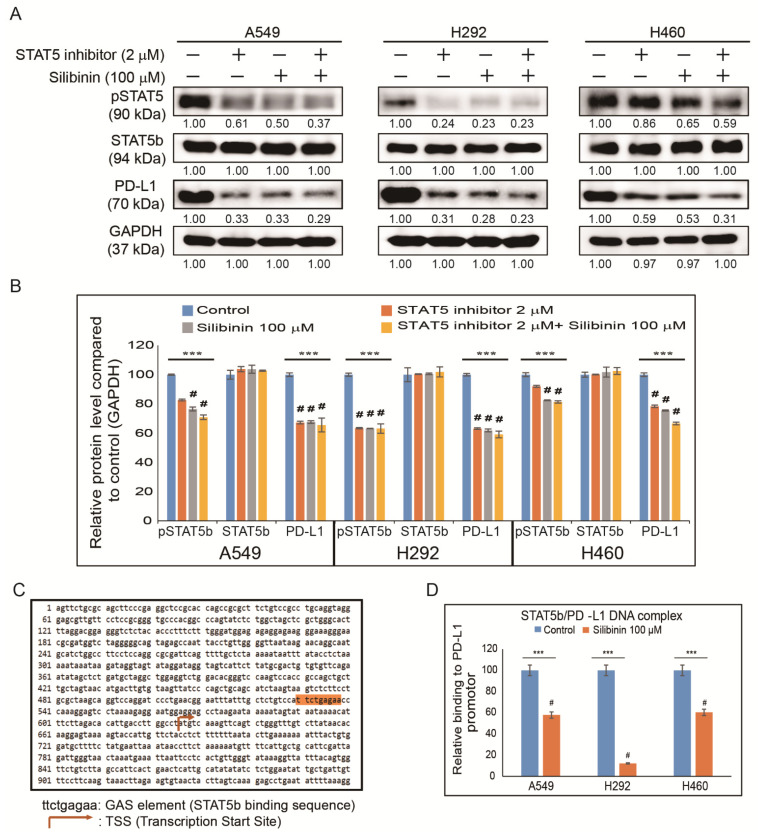
Silibinin inhibited STAT5 binding to the PD-L1 promoter. (**A**) Western blotting analysis showed that 2 µM STAT5 inhibitor suppressed pSTAT5b and PD-L1 in A549, H292, and H460 cells, and its combination with 100 µM silibinin has also shown the same inhibitory effect. (**B**) Representative expression of proteins was determined by densitometry and normalized to GAPDH. Controls were set to 100. Experiments were repeated thrice to confirm the data. *** *p* < 0.001 (ANOVA); # *p* < 0.001 vs. control. (**C**) Sequence of the PD-L1 promoter (available online: https://www.ncbi.nlm.nih.gov/nuccore/NC_000009.12?report=genbank&from=5450542&to=5470554 (accessed on 25 June 2021)). A GAS element (ttctgagaa) present in the PD-L1 gene (nucleotide sequence 529–538) is highlighted. (**D**) ChIP assay analysis after A549, H292, and H460 cells were incubated with 100 µM silibinin for 48 h showed suppression in STAT5 binding to PD-L1 in A549, H292, and H460 cells. The relative binding of STAT5 to the PD-L1 gene promoter was expressed as a percentage of control. Statistical analysis was performed using Student’s *t*-test (*** *p* < 0.001). # *p* < 0.001 vs. control.

**Figure 7:**
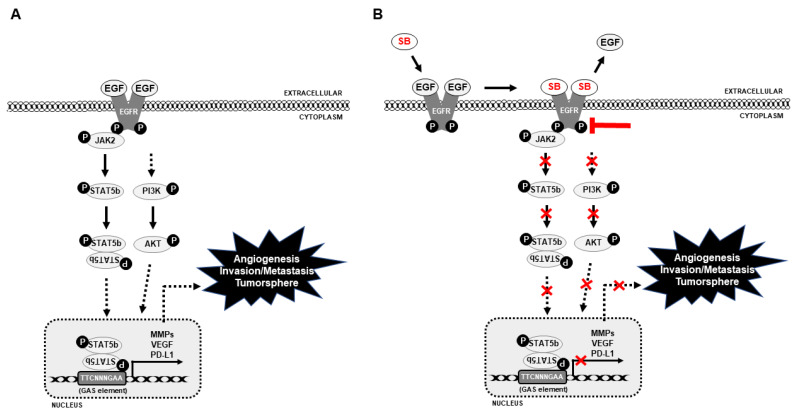
Molecular regulatory mechanism behind the anticancer activity of silibinin in NSCLC cells. (**A**) Molecular mechanism of NSCLC without silibinin and (**B**) silibinin exhibits an inhibitory effect on EGFR-mediated PI3K/AKT and JAK2/STAT5 signaling pathway and thereby PD-L1 inhibition (SB: Silibinin).

As the AKT pathway is well known to control PD-L1 expression in NSCLC [19], it was assumed that STAT5 might have a direct connection with PD-L1 expression. To check the relationship between STAT5 and PD-L1 in NSCLC cells, STAT5 expression was inhibited using a specific STAT5 inhibitor (Figure 6A). The results indicated a significant inhibition in pSTAT5 and PD-L1 protein expression in silibinin-treated samples and its combination with STAT5 inhibitor samples (Figure 6B). Whether inhibition in pAKT expression could suppress MMP2 expression was assessed and to check this relationship in NSCLC cells, AKT was suppressed using a specific AKT inhibitor. The results indicated a significant inhibition in pAKT and MMP2 protein expression in silibinin-treated samples and its combination with AKT inhibitor samples (Figure S2) [12]. As clearly indicated in previous results, shown in Figure 5C, it was assumed that through EGFR/JAK2/STAT5 signaling STAT5 could translocate into the nucleus and bind to the PD-L1 promoter region to facilitate immune escape and promote metastasis. Using a gamma interferon activation site (GAS) element considered as the binding site of STAT5, a binding site of STAT5 (ttctgagaa) was found in the PD-L1 promoter region (Figure 6C). To confirm the binding site of STAT5 in the PD-L1 promoter region, primers specific to the PD-L1 genome were designed, and its binding was confirmed using ChIP assay. Additionally, silibinin-treated samples showed significant inhibition of STAT5/PD-L1 binding (Figure 6D), clearly suggesting the role of the STAT5/PD-L1 signaling cascade in the anticancer activity of silibinin against NSCLC cells that could suggest silibinin as an immunotherapeutic drug against lung cancer cells. Altogether, silibinin can inhibit cancer hallmarks against NSCLC cells and also inhibited PD-L1 expression through EGFR-mediated PI3K/AKT and JAK2/STAT5 signaling mechanisms (Figure 7).

## 4. Discussion

Natural compounds are one of the target therapies for various cancers with fewer side effects [31,32]. Silibinin is one of the natural compounds shown to be efficient in anticancer treatment due to its ability to inhibit the proliferation, migration, and invasion of triple-negative breast cancer (TNBC) cells [33]. Silibinin also indicated pSTAT3 inhibitory effects in preclinical models [34] and inhibition of NSCLC metastasis by targeting the EGFR/LOX pathway [30]. Although many studies suggested the anticancer ability of silibinin and its ability to inhibit PD-L1 expression in nasopharyngeal cancer [35], its possible mechanism behind the STAT5 pathway and PD-L1 expression in NSCLC are still unknown.

A drug could be considered for anticancer studies if it can act on some of the hallmarks of cancer, including proliferative signaling, resistance to cell death, evasion of growth suppressors, induction of replicative immortality, initiation of angiogenesis, and stimulation of invasion and metastasis [19]. The anticancer ability of silibinin against lung cancer is well known [36,37] and our results also showed that increasing concentrations of silibinin induced cell death in NSCLC cells (A549, H292, and H460) [28].

Mutation in tumor suppressor genes can change the expression levels and activities that might lead to tumorigenesis, resulting in cell cycle and apoptosis [38]. Silibinin as a natural compound can induce cell cycle arrest G1 phase in AsPC-1 cells and apoptosis in pancreatic cancer cells by activating caspase-3/8/9 [39,40]. In this study, experiments were conducted to check whether silibinin has the same effect on NSCLC, and data indicated that silibinin induced G0/G1 arrest in all cell lines (A549, H292, and H460) and there was a notable downregulation of cell cycle proteins (CDK4, cyclin D1, and cyclin E). Silibinin also induced the expression of tumor suppressor proteins (p21 and p27) and tumor apoptosis. Therefore, these results suggest silibinin has a strong background for anticancer activity.

For a tumor to grow, it needs a sufficient oxygen supply; however, this may be lacking due to the increase in tumor size. This will cause hypoxic conditions that generate hypoxia-inducible factor-1α to activate VEGF to allow oxygen to reach the tumor microenvironment through blood supply [41]. After the primary tumor is settled, it begins to move to other parts of the body and make new tumors. Tumor cells will invade the bloodstream and migrate through blood vessels to spread the tumor, which is considered the initial step in metastasis [42]. The results indicated a decrease in in vitro angiogenesis through the inhibition of tube formation by silibinin in HUVECs. Silibinin breaks the tube formation, causing the absence of vessel formation responsible for tumor oxygen supply during hypoxic conditions. Molecular mechanism analysis of silibinin in HUVECs also indicated an inhibition in VEGF, and both pSTAT5 and AKT expression as STAT5 play an important role in tumor angiogenesis through the regulation of VEGF activity [43]. The inhibition of tumor migration and invasion in A549, H292, and H460 cells by silibinin has clearly suggested its role in suppressing tumor metastasis.

Studies have shown that cancer cells can form a sphere when in vitro cultured in a serum-free medium [44,45]. Other studies have also shown the inhibitory effect of natural compounds on tumorsphere formation, such as salanomycin reduced OCT4, NANOG, and SOX2 expression in A546 cells [46]. Curcumin was found through the JAK2/STAT3 pathway to reduce tumorsphere growth in H460 lung cancer cells in in vivo and in vitro studies [47]. Data from experiments clearly indicated that silibinin could suppress tumorsphere formation and its specific CSC marker genes (*SOX2*, *OCT4*, and *NANOG*), suggesting the ability of silibinin to suppress mutation in CSC signaling and for use in chemotherapy, as it can target CSC to prevent cancer recurrence.

In general, molecular signaling begins by receptor binding, and EGFR is a famous receptor in cancer formation where its mutation may lead to tumorigenesis. As an intracellular tyrosine kinase domain necessary for signaling pathways [48], EGFR overexpression is detected in most NSCLC cells and its regulation could be essential to manage tumor progression. The results from experiments on recombinant EGF showed that silibinin could bind to EGFR and suppress EGFR phosphorylation and thereby block its downstream signals, also resulting in the suppression of JAK2 phosphorylation. A member of the Janus kinase family, JAK2 is involved in different cell signaling reactions [49], and this interaction with other proteins, such as STAT and PI3K, may cause cell death or tumor formation [50,51], mediate MMP proteins [33], and enhance PD-L1 regulation, which has been proven to have a role in tumor metastasis and immune escape [52]. Therefore, the results demonstrated that silibinin could drive the downregulation of these transmembrane proteins signals that finally regulated MMP2 and PD-L1 expression in NSCLC.

The relationship between AKT signaling and PD-L1 expression is well known in NSCLC cells [19]. However, studies on the connection between STAT5 and PD-L1 are still a hot topic in NSCLC cells, as the JAK/STAT pathway is also a major pathway involved in NSCLC tumor progression. The presence of the GAS element in the PD-L1 genome gives an idea of the STAT binding site in the PD-L1 promoter region so that STAT5b could act as a transcription factor for PD-L1. This binding activity was confirmed by ChIP assay analysis, and treatment with silibinin showed significant inhibition of STAT5/PD-L1 binding. These results suggested the direct relationship between STAT5b and PD-L1 and a possible candidate drug for immunotherapy by targeting PD-L1 expression and thereby inhibiting the immune escape mechanism. Although the conclusion of the study is supported by the obtained results, the absence of in vivo experiments could be considered as a limitation of this study.

## 5. Conclusions

Altogether, silibinin inhibits NSCLC proliferation by mediating the JAK2/STAT5 and PI3K/AKT pathways that induce G0/G1 phase cell cycle arrest and apoptosis and inhibit tumorsphere formation. Through regulation of VEGF expression, silibinin exhibited an inhibitory ability against tumor angiogenesis and invasion and acts on PD-L1 downregulation, which is an immune response suppressor. Additionally, STAT5 has a direct connection with PD-L1 expression, and silibinin suppresses STAT5/PD-L1 complex formation, which can be used as a therapeutic target for immunotherapy and CSC-mediated targeted therapy.

## Data Availability

The data presented in this study are available on request from the corresponding author.

## Supplementary Materials

The following are available online at https://www.mdpi.com/article/10.3390/cells10071632/s1. Figure S1. Silibinin prevented NSCLC cell proliferation. (A) MTT assay illustrates cell proliferation inhibition of A549, H292, and H460 cells depending on silibinin concentrations for 48 h. Values are the mean ± SEM of three independent experiments performed in triplicate (n = 3). Controls were set to 100. Experiments were repeated thrice to confirm the data. *** *p* < 0.001 (ANOVA); # *p* < 0.000 vs. control. (B) Silibinin caused nuclear deviations in NSCLC cells. Microscopic observation showed abnormal nucleus formation by 50 and 100 µM silibinin in A549, H292, and H460 cells obtained from DAPI staining (1st row) and bright field (2nd row). Representative photographs are presented. Scale bar, 200 μm. Figure S2. Silibinin inhibited AKT-mediated MMP2 expression in NSCLC. Western blotting analysis showed that 2 µM AKT inhibitor suppressed pAKT and MMP2 in A549, H292, and H460 cells, and its combination with 100 µM silibinin has also shown the same inhibitory effect. Representative expression of proteins was determined by densitometry and normalized to GAPDH. Controls were set to 100. Experiments were repeated thrice to confirm the data. *** *p* < 0.001 (ANOVA); # *p* < 0.001 vs. control. Table S1. qPCR primer sequences and annealing temperature.
