# Supplementary material for: Silibinin Regulates Tumor Progression and Tumorsphere Formation by Suppressing PD-L1 Expression in Non-Small Cell Lung Cancer (NSCLC) Cells
Source: Cells. 2021 Jun 29;10(7):1632. doi: 10.3390/cells10071632 (PMC8307196; doi:10.3390/cells10071632)
Supplement: Supplementary file 1 [file cells-10-01632-s001.zip › Table S1.pdf]

Table S1. q-PCR primer sequences and annealing temperature.

**Sl No**	**Gene**	**Annealing temperature (°C)**	**Sequence (5` - 3`)**
**1**	SOX2	58	F- 5′-aaccagcgcatggacagtta-3′<br>R- 5′-cgagctggtcatggagttgt-3′
**2**	OCT4	58	F- 5′-caaagcagaaaccctcgtgc-3′<br>R-5′- aaccacactcggaccacatc-3′
**3**	NANOG	58	F- 5′-accagtcccaaaggcaaaca-3′<br>R- 5′-tctgctggaggctgaggtat-3′
4	*MMP2*	58	F: 5′-tgatggcatcgctcagatcc-3′<br>R: 5′-ggcctcgtataccgcatcaa-3′
5	*MMP3*	58	F: 5′-cacagacctgactcggttcc-3′<br>R: 5′-aggttctggagggacaggtt-3′
6	*MMP9*	58	F: 5′-ggacaagctcttcggcttct-3′<br>R: 5′-tcgctggtacaggtcgagta-3′
7	*VEGF*	58	F: 5′-aggagggcagaatcatcacg-3′<br>R: 5′-caaggcccacagggattttct-3′
8	*PD-L1*	58	F: 5′-tgccaggcattgaatctaca-3′<br>R: 5′-ggcctatttcctcctcttgg-3′
9	*GAPDH*	58	F: 5′-cccactcctccacctttgac-3′<br>R: 5′-tcctcttgtgctcttgctgg-3′
10	*PD-L1* (ChIP assay)	58	F: 5′-cccagctgcagcatctaagt-3′<br>R: 5′-aggccaaggtcaatgtgtct-3′
